# Body composition variations between injured and non-injured professional soccer players

**DOI:** 10.1038/s41598-022-24609-4

**Published:** 2022-12-01

**Authors:** Francisco Martins, Cíntia França, Ricardo Henriques, Andreas Ihle, Krzysztof Przednowek, Adilson Marques, Hélder Lopes, Hugo Sarmento, Élvio Rúbio Gouveia

**Affiliations:** 1 Department of Physical Education and Sport, University of Madeira, Funchal, Portugal; 2 LARSYS, Interactive Technologies Institute, Funchal, Portugal; 3 Research Center in Sports Sciences, Health Sciences, and Human Development (CIDESD), 5000-801 Vila Real, Portugal; 4 Marítimo da Madeira – Futebol, SAD, Funchal, Portugal; 5 Department of Psychology, University of Geneva, 1205 Geneva, Switzerland; 6 Center for the Interdisciplinary Study of Gerontology and Vulnerability, University of Geneva, 1205 Geneva, Switzerland; 7 Swiss National Centre of Competence in Research LIVES—Overcoming Vulnerability: Life Course Perspectives, 1015 Lausanne, Switzerland; 8 Institute of Physical Culture Sciences, Medical College of Rzeszów University, Rzeszów University, Rzeszów, Poland; 9 Centro Interdisciplinar de Estudo da Performance Humana, Faculdade de Motricidade Humana, Universidade de Lisboa, 1499-002 Lisbon, Portugal; 10 ISAMB, University of Lisbon, 1649-020 Lisbon, Portugal; 11 Research Unit for Sport and Physical Activity (CIDAF), Faculty of Sport Sciences and Physhical Education, University of Coimbra, Coimbra, Portugal

**Keywords:** Health care, Risk factors, Physics

## Abstract

Professional soccer is characterized by its physical demands, making players’ exposure to high injury risks a growing problem. It is crucial to study the factors associated with injuries in professional soccer. This study aimed to investigate the relationship between age, body composition, and others variables related with the injury profile of professional soccer players of a specific Portuguese team. Also, it analyzed the impact of the injury profile on soccer’s variations in body fat (BF%), skeletal muscle mass (SMM) and total body water (TBW) throughout the season. The sample comprised 31 male professional soccer players competing in the First Portuguese Soccer League. Older players had a higher prevalence of muscular injuries. Midfielders and forwards showed the highest number of muscular injuries during the season being quadriceps the most affected zone. Considering players’ BF% [Wilks’ Lambda = 0.42, F (7, 23) = 4.61, p = 0.002, r = 0.58], SMM [Wilks’ Lambda = 0.59, F (6, 23) = 2.70, p = 0.039, r = 0.41] and TBW [Wilks’ Lambda = 0.54, F (7, 23) = 2.80, p = 0.029, r = 0.46] there was a substantial main effect for the assessments performed throughout the season and the injury status. Age assumes relevance in explaining the injury profile. The impact of the injury profile on soccer's variations in BF%, SMM and TBW throughout the season must be analyzed considering the clinical relevance.

## Introduction

At a professional level, soccer is characterized by its high-speed and intense actions^1,2^, which may contribute to a higher players’ exposure to the risk of injury^3^. According to the literature, an injury is an event that occurred during a scheduled training session or match, resulting in an absence from the next training session or match^4^. Besides being prejudicial to the players’ careers, the injury occurrence also harms the overall team’s performance^3,5–9^. Muscular injuries are the most frequent in professional soccer players, accounting for 20–37% of all time-loss injuries at this competitive level^4,10–13^. The reasons that lead players to muscular damage are poorly understood. Few investigations, including senior professional players and providing precise information on muscular injuries needed for this purpose, have been conducted^14^. The differences between injured and non-injured soccer players regarding body composition variables have been analyzed by some authors, demonstrating their relevance as a risk factor for injury in soccer^15–19^. A study conducted with collegiate soccer players from Division I reported a significant increase of 0.5% in body fat percentage (BF%) across an entire season^16^. Another investigation of 398 players from eight age and skill-level groups, concluded that BF% presented significant differences between injured and non-injured players^17^.

Although most studies recommend and follow to focus on injury incidence, it is believed that focusing only on this variable may lead to an incomplete reading of the sports injury phenomenon^20^. Therefore, the aims of this study were twofold: (1) to investigate the muscular injuries occurrence of a portuguese professional soccer team throughout a season, considering non-modifiable (i.e. age) and modifiable variables, such as body composition, the prevalence of injuries, exposed time in training and matches, players’ sectorial position, consequences of injuries, and match periods occurrence, and (2) to analyze variations in the body composition variables between injured and non-injured players of a Portuguese professional soccer team throughout the season.

## Methods

### Participants

Thirty-one professional male soccer players participated in this study (age: 25.5 ± 3.4 years, height: 182.1 ± 6.9 cm, body mass 79.1 ± 7.9 kg). Twenty-three participants had the right lower limb as dominant, and the remaining eight had the left lower limb as dominant. All participants competed in the First Portuguese League during the 2020/2021 season. The optimal sample size calculation was calculated using G*Power3^21^. In the first analyses, a priori Mann–Whitney test indicated that a total sample size of 32 participants was needed to achieve 85% power to detect an interaction effect size of 0.70 at the 0.05 level of significance. In the second analysis, a priori, repeated-measures ANOVA, within-between interaction, eight measures, correlation among repeated-measures of 0.9, indicated that a total sample size of 30 participants was needed to achieve 95% power to detect an interaction effect size of 0.10 at the 0.05 level of significance.

All procedures applied were approved by the Ethics Committee of the Faculty of Human Kinetics, CEIFMH N° 34/2021. The investigation was conducted following the Declaration of Helsinki, and informed consent was obtained from all participants.

### Body composition

Body composition variables were assessed using hand-to-foot bioelectrical impedance analysis (InBody 770, Cerritos, CA). Height was measured to the nearest 0.01 cm using a stadiometer (SECA 213, Hamburg, Germany). The measurement occurred in the early morning, with participants fasting and using only their underwear. During the assessment, participants were barefoot, standing with both arms 45° apart from the trunk, and with both feet bare on the spots of the platform. Twenty-six evaluations of body composition were performed during the season. The body mass, BF%, and total body water (TBW), skeletal muscle mass (SMM), phase angle, intra cellular water, and extra cellular water were the variables retained for analysis. The reliability analysis showed an excellent internal consistency (Cronbach’s alpha coefficient was 0.99).

### Injury Report

This study followed the recommendations of the Union of European Football Associations (UEFA) for epidemiological investigations. An injury was defined as an event during a scheduled training session or match, resulting in an absence from the next training session or match^4^. Injury records during the season, including training and competitive moments, were made daily by the clinical department. The variables analyzed were type, zone, exposure, incidence, severity, and occurrence regarding injuries. Additionally, if an injury occurred during a match, the minute of the event was registered. All the injuries throughout the season were recorded. Two groups were built according to the injury status: injured and non-injured.

Regarding the variables under analysis, the frequency of injuries by age corresponds to the number of injuries counted throughout the 2020/2021 season in the defined age intervals. The frequency of injuries by positional sector is characterized by the number of injuries that goalkeepers, defenders, midfielders and forwards contracted throughout the study. The type and zone of the injury are complementary variables that identify the part of the body that suffered structural and/or functional changes due to the contraction of an injury. Player exposure to injury was considered the time (in hours), of training and match moments, during which players were exposed to suffer an injury. The injury incidence was defined as the number of injuries contracted during sports activity, both in training and competitive moments, per 1000 h of exposure. The severity of the injury contemplates the period, in days, from the player's stoppage until resuming field work, with the consent of the clinical department. Finally, the injury occurrence was made weekly, and if a match situation caused it, the minute occurred was registered. At the end of the season, the injured players were followed until the end of their recovery period.

### Statistics

Descriptive statistics are presented as frequencies, proportions (%), and means ± standard deviations. Injury incidence was calculated as the number of injuries per 1000 playing hours. The injury burden was estimated as the number of days of absence per number of injuries. A Mann–Whitney *U* test was conducted to explore differences between groups in chronological age (CA) and body composition variables. Adjusted p-values by false discovery rate (FDR) were performed using the Benjamini–Hochberg procedure. The internal reliability between time points throughout the season was calculated using Cronbach’s alpha coefficient. A mixed ANOVA (between groups within subjects) was conducted to assess the impact of the injury profile on soccer’s body composition variables across eight periods throughout the season. The assumptions were verified, namely Levene’s Test of Equality of Error Variances and Equality of Covariance Matrices. All analyses were performed using the IBM SPSS statistics software 26.0 (IBM Corp. Armonk, NY, USA). The significance level was set at *p* ≤ 0.05.

### Ethical approval

This study was conducted according to the guidelines of the Declaration of Helsinki and approved by the Ethics Committee of the Faculty of Human Kinetics, (CEIFMH N° 34/2021), and followed the ethical standards of the Declaration of Helsinki for Medical Research in Humans (2013) and the Oviedo Convention (1997).

### Informed consent

Informed consent was obtained from all subjects involved in the study. Written informed consent has been obtained from all players to publish this paper.

## Results

### Muscular Injuries Characterization

The prevalence of muscular injuries was 29% throughout the season. On average, a player sustained 0.35 muscular injuries in the entire season. The exposure of all participating players was 587.6 h during matches, corresponding to 6.8 injury incidents per 1000 playing hours. They were assessed 8029 h during training sessions, corresponding to 0.87 injury incidence per 1000 playing hours.

Table 1 displays descriptive statistics for CA and body composition and the Mann–Whitney *U* test results at the beginning of the season. No significant statistical differences in non-modifiable and modifiable variables between injured and non-injured players were seen.

**Table 1:** Descriptive statistics for chronological age and body composition and results of Mann–Whitney *U* test.

Median (range)				Mann–Whitney *U* test		
Variables	All players (n = 31)	Injured (n = 9)	Non-injured (n = 22)	U	*p*	*p-adj*
CA (years)	25.8 (24.5–28)	27.4 (26.5–29.2)	25.4 (22.3–27.1)	44.000	0.017	0.527
Height (cm)	183 (178.1–187.3)	184.1 (178–186.1)	181.3 (179–188.1)	97.000	0.931	1.000
BM (kg)	79.8 (74.5–84.2)	79.8 (76.4–83.1)	79.5 (73.8–84.2)	92.000	0.761	1.000
BF (%)	10.6 (9.4–12)	10.6 (9.6–14.1)	10.5 (9.2–11.8)	85.000	0.542	1.000
SMM (kg)	40.5 (30.4–49.8)	40.5 (32.0–44.8)	41.1 (30.4–49.8)	98.000	0.965	1.000
Phase angle	7.1 (6.3–7.7)	7.1 (6.4–7.5)	7.2 (6.3–7.7)	87.500	0.615	1.000
TBW (l)	52.1 (48.2–54.3)	52.1 (50–54)	52.1 (48.2–54.3)	99.000	1.000	1.000
ICW (l)	32.6 (24.8–39.8)	32.6 (26.1–35.9)	33.0 (24.8–39.8)	98.500	0.983	1.000
ECW (l)	19.1 (14.5–22.6)	19.0 (15.0–20.9)	19.2 (14.5–22.6)	96.000	0.915	1.000

*95% CI* 95% confidence interval, *CA* chronological age, *BF*% body fat percentage, *TBW* total body water, *BM* body mass, *SMM* Skeletal muscle mass, *ICW* intra cellular water, *ECW* extra cellular water.

Table 2 presents descriptive statistics according to the players’ sectorial position. The midfielders and forwards showed the highest number of muscular injuries during the season, corresponding to 66.6%.

**Table 2:** Descriptive statistics for the sectorial position.

Variables	All players (n = 31)	Injured (n = 9)	Non-injured (n = 22)
Goalkeeper	3 (9.7%)	1 (11.2%)	2 (9.1%)
Defender	11 (35.5%)	2 (22.2%)	9 (40.9%)
Midfielder	7 (22.6%)	3 (33.3%)	4 (18.2%)
Forward	10 (32.2%)	3 (33.3%)	7 (31.8%)

Table 3 shows the number of muscular injuries according to the body’s zone. The quadriceps were the most affected zone, with five injuries reported, leading to the highest number of days of absence from matches and training sessions. The muscular injuries in the hamstrings and adductors mainly occurred during match situations. However, the data shows that the adductors' injuries led to longer stoppages than those sustained in the hamstring area.

**Table 3:** Incidence of muscular injuries according to the body’s zone.

	n	Training	Match	Total absence, d	Injury burden^a^	Missed trainings	Missed matches
All Muscular injuries	11	7	4	172	15.6	122	22
Quadriceps	5	5	0	115	23	82	15
Hamstrings	3	1	2	16	5.3	11	1
Adductor	3	1	2	41	13.7	29	6

^a^Injury burden is expressed as the number of days of absence/number of injuries.

**Figure 1:**
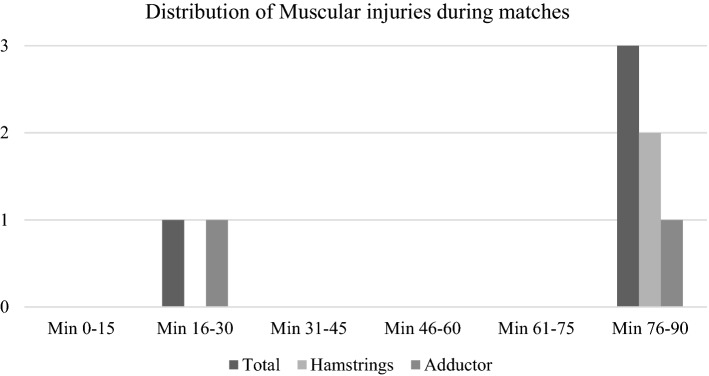
Distribution of muscular injuries during a match over an entire season.

Figure 1 illustrates the injuries that occurred during match periods (each 15 min). Most of the injuries were observed during the last 15 min of the match, particularly in the hamstrings and adductor areas.

### Body composition variations

**Figure 2:**
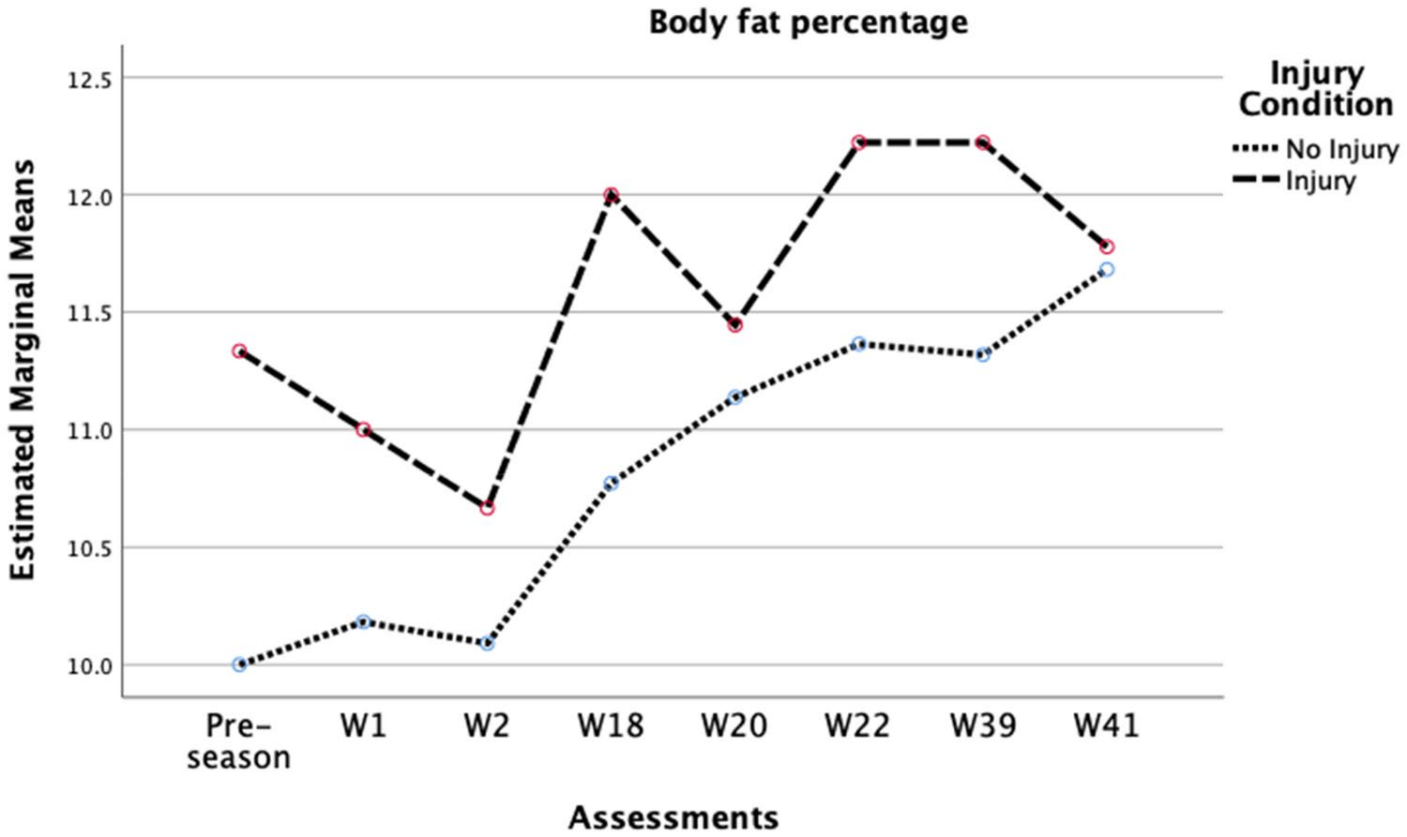
Variation in players’ body fat percentage across the season 2020/2021.

**Figure 3:**
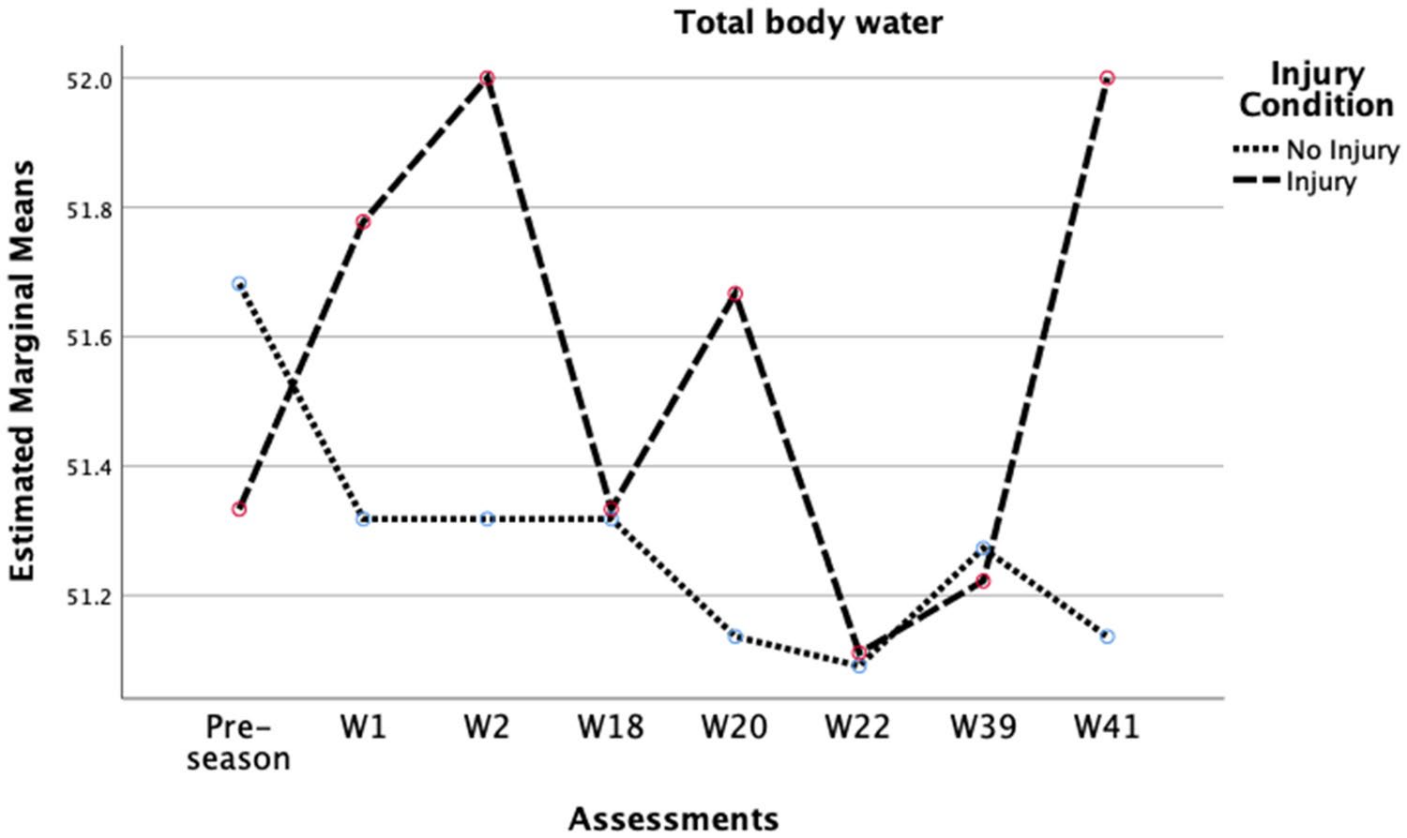
Variation in players’ total body water across the season 2020/2021.

**Figure 4:**
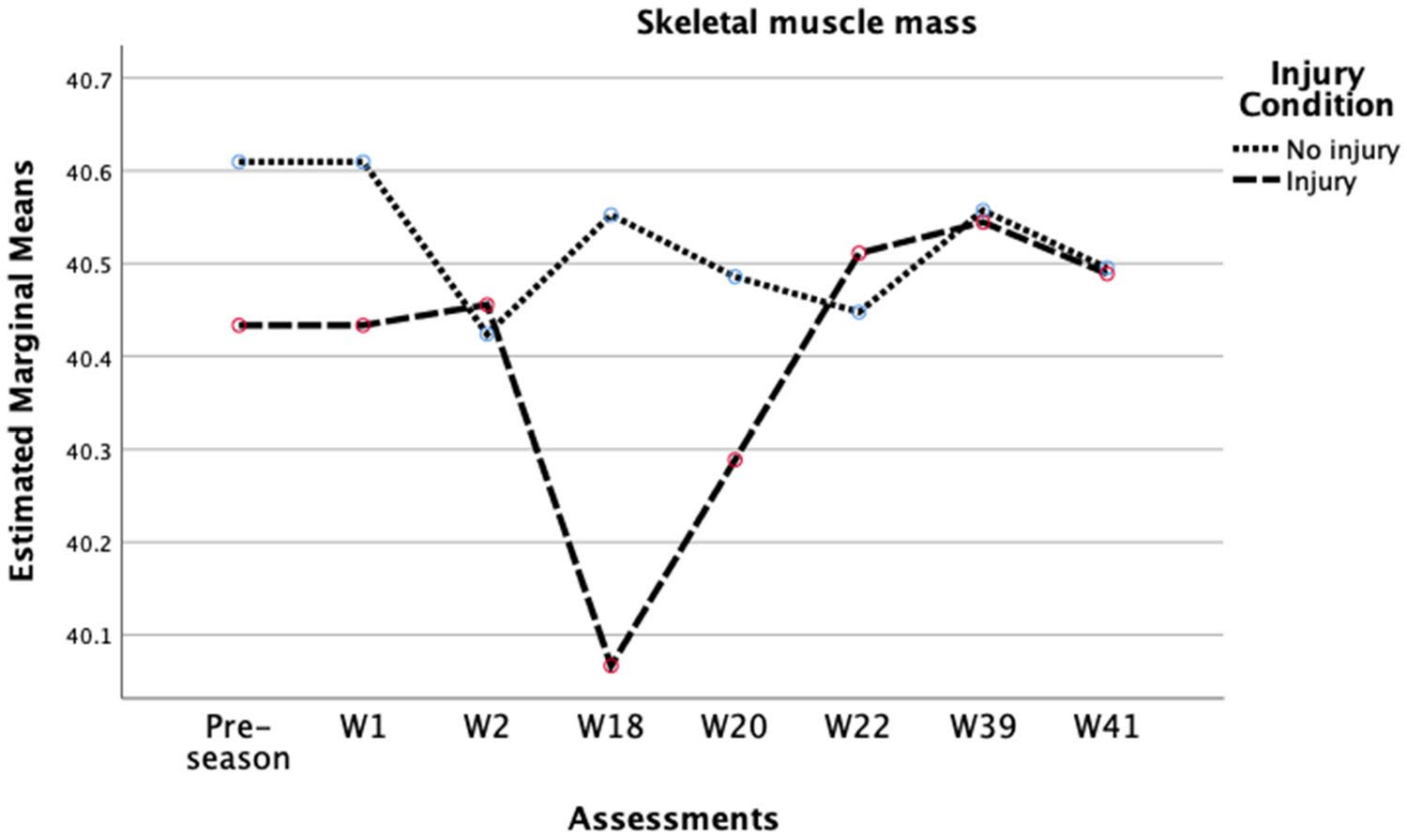
Variation in players’ skeletal muscle mass across the season 2020/2021.

Considering players’ BF% [Wilks’ Lambda = 0.42, F (7, 23) = 4.61, p = 0.002, r = 0.58, moderate effect size] (Fig. 2), TBW [Wilks’ Lambda = 0.54, F (7, 23) = 2.80, p = 0.029, r = 0.46, moderate effect size] (Fig. 3), and skeletal muscle mass, [Wilks’ Lambda = 0.59, F (6, 23) = 2.70, p = 0.039, r = 0.41, moderate effect size] (Fig. 4), there was a substantial main effect for the assessments performed throughout the season and the injury status, suggesting that the range pattern of BF%, TBW, and skeletal muscle mass throughout the season, is different between injured and no-injured players. No other main effects were seen in phase angle, intra-cellular water, or extra-cellular water. Besides that, with the internal reliability test between the eight time points throughout the season in Fig. 2 (α = 0.98), the results demonstrate that the most significant difference in BF% from one moment to another, in both groups, was from week 2 (10.2 ± 2.4%) to week 18 (11.0 ± 2.6%), with a difference of 0.8%. Regarding TBW, the internal reliability was α = 0.99 (Fig. 3), being verified that the most significant difference from one moment to another in the injured players was between week 2 (51.8 ± 4.3 L) and week 18 (51.3 ± 4.3 L), with a difference of 0.5 L. In non-injured players, the main variation occurred between the pre-season (51.6 ± 5.7 L) and week 1 (51.3 ± 5.6 L) of competition, with a difference in this variable of 0.3 L. In skeletal muscle mass, the internal reliability was α = 0.99 (Fig. 4), with the most significant difference between assessments in the injured players seen between week 2 (40.5 ± 3.8 kg) and week 18 (40.1 ± 3.6 kg), with a difference of 0.4 kg. In non-injured players, the main variation occurred between week 1 (40.6 ± 4.6 kg) and week 2 (40.4 ± 4.6 kg) of competition, with a difference in this variable of 0.18 kg.

## Discussion

This study aimed to specify the muscular injuries occurrence throughout a season, considering non-modifiable (i.e. age) and modifiable variables, such as body composition, the prevalence of injuries, exposed time in training and matches, players’ sectorial position, consequences of injuries, and match periods occurrence. Also, it was analyzed the impact of the injury profile on soccer’s variations in BF%, TBW, and SMM throughout the season. In this study, older players presented a higher number of muscular injuries than their younger peers. These results would be expected since literature has described the higher risk of injury among older players^14,15,19^. There are well-known age-related changes in biological and physiological components and historical events related to playing soccer that makes older players more vulnerable to injury.

Regarding body composition, no significant differences were observed between injured and non-injured players at the beginning of the season, which is expected since this is a very homogenous group of professional soccer players. In addition, there was a monitoring process implemented throughout the season on this team, i.e. assessments in body composition being performed weekly, that would help to control this physical parameter. Although no substantial differences were observed in our study for body composition variables between injured and non-injured players, it is still crucial to emphasize the importance of monitoring those variables across the season. Indeed, the literature has pointed out body composition, particularly BF%^16^, SMM^22^, and phase angle^23^ as a crucial component in enhancing physical capacities. The detrimental effect of BF% on jumping, sprinting, and change of direction tasks has been described in several sports contexts^24,25^. In this study, the prevalence of muscular injuries was 29% throughout the season. In line with our findings, some studies state that 20–37% of the injuries that occur at a professional soccer level are muscular injuries^4,10,11,13^. Indeed, muscular injuries represent the most common injuries that professional soccer players must face throughout their careers^3,14,26^. On the other hand, our analysis found-out a mean of 0.35 muscular injuries per player over the investigated period. In a study designed between 2001 and 2009 with 2299 professional soccer players, authors described a mean of 0.6 muscular injuries per player over one season^14^. Another study conducted across three consecutive seasons with 227 professional male youth soccer players aged 16.8 ± 3.1 years reported 0.92 muscular injuries per player across the study period^12^. Overall, previous results on this topic are higher than those in our study. This could be related to the different training methods used by teams and the type of competition that they were involved in, but also to intrinsic biological, physiological, and even physhological variables. Due to the multidimensional negative impact of the injuries of soccer players, this data may raise awareness among sports agents and coaches to consider the injury assessment as an essential tool in the players’ monitoring and implement preventive injury measures during the season.

This study suggests that players were exposed to 587.6 playing hours during matches, with a 6.8 injury incidence per 1000 h. Players were submitted to 8029 playing hours in training sessions, corresponding to a 0.87 injury incidence per 1000 h. Past studies have described an average injury incidence of 6.8^12^ and 8.7^14^ per 1000 playing hours in match situations. However, the injury incidence during training sessions was substantially higher than our findings, corresponding to 3.20/1000h^12^ and 1.37/1000h^14^. Indeed, these results should be related to each team’s individual training methods and the players’ characteristics, making comparisons difficult. For example, the professional soccer team analyzed in our study was exposed to a preventive injury program, mainly based exercises of mobility, myofascial, proprioceptive, plyometric, eccentric strength, bilateral and unilateral strength, isometric and dynamic core. These sessions varied between the field and the gym, and the athletes performed them in 2 sets with 4 to 8 repetitions, lasting 15–25 min.

Additionally, despite the exposure time to training being considerably higher than the exposure time to match, our analysis indicates a higher risk of injury occurrence in the match than in training situations. This conclusion is supported by previous literature^3,12,26–28^, and reinforces the need of monitoring the players’ external and internal load during the season (in training and the matches), with the purpose of identified situations of overload. This information might be crucial to improving players’ performance and reducing the injury risk, particularly during matches.

Regarding the players’ sectorial position, the midfielders and forwards showed the highest number of muscular injuries compared with their peers, which is consistent with previous research on this topic^3,28^. In contrast, the goalkeeper position was the least affected, which should be related to their lower exposure to situations of physical contact and lower level of physical demands while playing^3^.

Concerning the consequences of muscular injuries over a season for a typical professional soccer team, the most affected areas were the quadriceps, the hamstrings, and the adductors^14^., reported that those were the three most frequent muscular injuries in a study made across nine years, comprising 2299 players from 24 clubs selected by UEFA. Our study has found 11 muscular injuries across a sports season^14^., stated that a team with 25 players could expect about 15 muscular injuries per season, which is a higher value than the one found in our study. On average, a player lost two weeks of training and competition after suffering a muscular injury, losing an average of 11 training sessions and two matches, consistent with previous studies^14^.

According to the literature, the injury occurrence was more prevalent at the match’s end^12,14^. Our results corroborate previous findings since 75% of the muscular injuries in matches occurred in the last 15 min. Indeed, fatigue would be expected to have a higher injury prevalence at the end of the match’s second half. This information brings crucial insights into the soccer training process. It may call for awareness among sports agents and coaches from three perspectives. One regards the need to approximate the training sessions to match reality in terms of intensity to overcome the adverse effects of fatigue; the second one underlines the need to quantify, objectively (i.e. using GPS monitorization), the training and matching locomotor/physiological stimuli in real-time; the third one is the fact that coaches should take this information into account, so that they can choose the most appropriate in-game moments to make substitutions. These three perspetives would help teams’ staffs to identify soccer players in overload risk and make them stop before the injury occurrence. Throughout the analyzed season, strength and speed exercises were included at the end of training sessions as a strategy to help delay athlete’s fatigue, as this arises from decreasing their strength levels.

Finally, this study analyzed the impact of the injury profile on soccer’s variations in BF%, SMM, and TBW throughout the season. Our results sustain that the pattern of injured players throughout the season is much more unbalanced than the pattern of non-injured players in those variables. A study contemplating 398 players from eight different age and skill-level groups reported that only BF% showed significant differences between injured and non-injured players^17^. In another study on a Division I team, where 53 soccer players (20.3 ± 1.3) were analyzed during an entire season, the authors described a substantial increase of 0.5% in BF%^16^. Although past literature on the assessment of body composition considering the injury condition is scarce, previous findings are consistent with our results, particularly regarding the increase of BF% during the season. Despite this statistically significant increase of 0.8% seen in our study, between week 2 and week 18, probably due to the high internal reliability of the body composition assessments (α = 0.98), we must be aware that in terms of clinical significance, an increase of 0.8% may not represent a substantial increase in this population.

In terms of SMM, it was observed that the results achieved in this variable by the non-injured players were remarkably stable and with minimal variations throughout the sports season analyzed. On the contrary, we observed a greater oscillation in the values achieved by the group of players who were injured throughout the sports season. Indeed, the process of recovery from sports injuries restricts the movement and stimulation of the injured limb, leading to muscle disuse^29–31^. Moreover, the consequences of this period will be even more evident in the rehabilitation time of the athlete for the return to competition^31–33^. Scientific evidence reinforces that increasing protein intake, type and timing of dietary protein ingestion can combat the loss of skeletal muscle mass throughout the shutdown period^34–36^. Also, neuromuscular electrical stimulation is important to stimulate involuntary muscle contractions and assist in the maintenance of muscle indexes in athletes^37,38^, so that there is not so much variation in this parameter of body composition. Thus, it proves once again the need for professional sports to adopt multidisciplinary measures in the areas of nutrition, injury prevention, and training methodologies. This will be a crucial upgrade in sporting and economic terms, since, in professional terms, the economic cost to a club of having its players out of competition is considerable^31,39,40^.

Regarding TBW, although non-significant, the injured group showed higher values of TBW compared to the non-injured group. We believe that clinically, this change is relevant for injury prevention in greater control of body composition variables throughout a sports season. In the sports literature, only few studies analyzed the relationship between TBW and the players' injuries, particularly among professional soccer players. Therefore, our study brings new insights to the soccer training context, reinforcing the importance of hydration of soccer players throughout the season. Relatively to the group of non-injured players, there was a decrease of Estimated Muscle mass values of Total Body Water. These players invariably ended up being exposed to more time, load and intensity of training and competition throughout the season. In physiological terms it is considered that the greater the physical effort of the players, the greater the need for the body tissues to absorb water, using it in the process of recovery and restructuring of the players, hydration levels. On the other hand, the players who suffered sports injuries throughout the season, were less exposed to the already mentioned training and playing conditions, and the physiological needs of one group and the other were inevitably different. The greater variation in total body water values in injured players portrays the balance between the moments of physical effort and the moments of recovery from sports injuries contracted throughout the analyzed sports season. Interestingly, an interaction between factors was observed only in week 22. After analyzing this situation in detail, it was concluded that in the two previous weeks (i.e. weeks 21 and 22), no player of this professional soccer team had suffered a sports injury, this being a valid justification for this interaction, since the homogeneity of the group in terms of number, time, load and intensity of training in the two weeks of work prior to this evaluation was observable.

There are some limitations to this study that need to be acknowledged. The sample size and the lack of data regarding players’ past injuries represent the main limitations of this study. However, these results bring important and specific practical implications for those involved in the professional soccer context.

Due to the homogeneity in body composition between professional soccer players, no differences were seen concerning the injury profile. Age is a non-modifiable related variable that assumes relevance in explaining the injury profile. This study’s prevalence of muscular injuries throughout the season follows the previous literature. This report confirms that during matches, the highest injury incidence occurred in the last 15 min. Also, regarding the sectorial position, midfielders and forwards presented the highest number of injuries compared with their peers. The impact of the injury profile on soccer's variations in BF%, SMM, and TBW throughout the season must be analyzed considering the clinical relevance. This investigation brings new insights to the soccer training context, reinforcing the importance of players’ monitoring process throughout a sports season, in body composition variables such as BF%, SMM, and TBW. The main contribution of the present study is to help team coaches to identify variable that can help forecast muscular injuries. In terms of practical implications, it represents that coaches and medical departments can anticipate and prevent injuries, making the player stop before injury occurs. With this early decision to spot the practice before the injury, the clubs can get important economical savings and gains because they will have the players more time to train and compete. Otherwise, sports agents and coaches should consider the injury assessment and introduce preventive injury programs while planning the sports season. These preventive programs should consider individual players' profiles.

## Data Availability

The data presented in this study are available upon request from the corresponding author.
